# Estimating Elasticity for Residential Electricity Demand in China

**DOI:** 10.1100/2012/395629

**Published:** 2012-09-10

**Authors:** G. Shi, X. Zheng, F. Song

**Affiliations:** ^1^Department of Techno-Economic Research, Development Research Center of the State Council, Beijing 10010, China; ^2^School of Economics, Renmin University of China, Beijing 100872, China

## Abstract

Residential demand for electricity is estimated for China using a unique household level dataset. Household electricity demand is specified as a function of local electricity price, household income, and a number of social-economic variables at household level. We find that the residential demand for electricity responds rather sensitively to its own price in China, which implies that there is significant potential to use the price instrument to conserve electricity consumption. Electricity elasticities across different heterogeneous household groups (e.g., rich versus poor and rural versus urban) are also estimated. The results show that the high income group is more price elastic than the low income group, while rural families are more price elastic than urban families. These results have important policy implications for designing an increasing block tariff.

## 1. Introduction

Since the open and reform policy initiated in 1978, China has managed to maintain rapid economic growth and emerged as the second largest economy in the world. In line with the rapid economic expansion of the past three decades, electricity demand has been growing substantially. Between 1980 and 2009, electricity consumption in China increased from 3,006 TWh to 37,032 TWh, at an annual rate of 8%. Over this period, residential electricity demand grew at a faster rate of 12%. The share of residential consumption over total electricity consumption went up from 3.5% in 1980 to 13.1% in 2009. However, China's per capita residential electricity consumption is still much lower than that of developed countries, accounting for about one-fifteenth of that of the US and one-seventh of that of Japan [1]. Given the continuing trend in income growth, modernization, and urbanization, growth in residential electricity demand is expected to remain high in China.

In the past, China's electricity industry has relied on making massive investments and increasing supply to meet the fast growing demand. The installed power capacity increased from 5.7 million kw in 1978 to 900 million kw in 2010. However, as the problems, such as energy security and environmental deterioration, become more and more severe in China, increasing energy supply is becoming more costly. To promote energy conservation and reduce the local pollutants and carbon emissions, the Chinese central government has set the national energy intensity reduction target for two consecutive five-year plans, which are 20% and 16% for the 11th and 12th five-year plans (FYPs), respectively. To reach this goal, some policies must be instigated. 

Given the side effects of supply policies, demand policies have been taking increasingly important roles in controlling electricity demand. Price policy, among these tools, has a particular role to play in energy conservation. In a competitive market, a rational agent would optimally reduce energy consumption in response to a higher price. However, China's energy sector is still heavily regulated by the government. Electricity pricing is completely controlled by the government. As pointed out by Lin and Jiang [1], China's residential electricity consumption is subsidized by industrial consumption and, thus, the residential tariff is even lower than the production costs. The twisted price signal encourages overconsumption and leads to a deterioration in the environment since most of China's electricity is generated by coal. Raising the residential electricity tariff to better reflect the real cost (both production costs and external costs) and to reduce the cross subsidy becomes more and more urgent as the electricity market reform moves forward. 

The policy effects of raising electricity tariffs are multifold. On one hand, raising electricity tariffs is believed to be an important measure to promote energy conservation and reduce emission. On the other hand, raising electricity tariffs will inevitably affect the welfare of the household, with differentiated effects on different social groups, such as rich versus poor or urban versus rural households. Quantitatively evaluating these policy effects needs good estimates of price and income elasticities of demand for electricity. Moreover, good estimates of price and income elasticities of demand for electricity are critical input parameters for many research studies. For example, the simulation results and subsequent conclusions of the computable general equilibrium (CGE) model, which is a popular analysis tool for evaluating energy and environmental policies, critically depends on the quality of the various energy demand elasticities.

Although estimating electricity elasticities has attracted much research effort since the first energy crisis of the 1970s, most of them focus on developed counties. J. Espey and M. Espey [2] reviewed 36 studies modeling residential electricity demand, among which only six studies focused on developing countries. Estimation of electricity elasticities based on rigid econometric analysis in China is especially scarce. The only study we found is that of Qi et al. [3], which estimated the price and income elasticities of residential electricity demand to be −0.15 and 1.06, respectively, using provincial level data between 2005 and 2007.

In this paper, price and income elasticities of Chinese residential electricity consumption are estimated for the first time based on data of a unique household survey. We specify the household electricity demand as a function of local electricity price, household income, and a number of social-economic variables (such as household size, age, and education of the household head and dwelling size). The objective of undertaking such estimation is to understand more deeply the key factors that influence electricity demand at household level in China.

We contribute to the literature in two aspects. First, to the best of our knowledge, this is the first paper that uses microlevel data to estimate the electricity demand elasticity in China. Aggregate level electricity consumption data are often employed to estimate electricity demand (e.g., among many other studies, [4–7]). However, the aggregate level data often lose much information at the individual level. Using microlevel data can better reflect the individual or household behavior and adds more details to our understanding of the consumers' response [8]. Second, we estimate the electricity elasticities across different heterogeneous household groups (e.g., rich versus poor and rural versus urban). This information contributes to the ongoing debate on how China's electricity market reform will affect different social groups and help design the new pricing scheme.

The rest of the paper is organized as follows.  Section 2 describes the empirical models we employed and is followed by the data and estimation results presented in Section 3. In Section 4, the results are discussed and conclusions are given.

## 2. Residential Demand for Electricity

### 2.1. Basic Model

Residential demand for electricity is a demand derived from the demand for a well-lit house, cooked food, hot water, and so on and can be specified using the basic framework of household production theory [5]. According to the theory, households purchase input factors to produce the final goods, which appear as arguments in the household's utility function. In our specific case, a household combines electricity and capital equipment to produce a composite energy commodity. 

Following the specification of [9, 10], a linear double-logarithmic form using income, electricity price, the price of alternative fuels, and a number of socioeconomic factors as independent variables is used in the empirical analysis of household electricity demand as follows:

(1) ln⁡Eit=β0+β1·ln⁡(PE_resp)+β2·ln⁡(Incomeit)+β3·ln⁡(PE_ngp)+β4·Zit+φt+ηit,

where *E*_*it*_ is electricity usage for household *i* at year *t*. PE_res_*p*_ is residential electricity price in province *p*, where household *i* lives. Income_*it*_ is real income of household *i* at year *t*. PE_ng_*p*_ is real price of liquefied natural gas in province *p*, which may be a complement to or substitute for electricity for households. Z_*it*_ is a set of control variables, including household dwelling area (HSIZE_*it*_), number of family members (FSIZE_*it*_), the age (AGE_*it*_), and years of education (EDU_*it*_) of the household head. *φ*_*t*_ is the year-fixed effect.

The key explanatory variables that influence household electricity demand are thus described in the model above. Household income and electricity price are two important economic variables that are assumed to determine household electricity demand. Since we adopt the double-logarithmic specification, the parameter estimates of *β*_1_ and *β*_2_ can be directly interpreted as price and income elasticity. 

Since electricity is not the only energy source for a household, electricity demand can also be influenced by the price of other alternative fuels. Therefore, we include the price of natural gas in the estimation of the demand functions. These are also included in the model in order to test the hypothesis of whether these fuels are in anyway complementary to or substitutes for electricity.

Several variables that represent household characteristics are included in the estimation model to account for the impact of the underlying preference of consumers of different backgrounds: (HSIZE_*it*_), number of family members (FSIZE_*it*_), the age (AGE_*it*_), and years of education (EDU_*it*_). We expect that a family which has more members or whose dwelling has a larger area may consume more electricity. How the age and education level of the household head will influence electricity consumption is an empirical question. The year-fixed effect is controlled to eliminate national trends, such as business cycles, that may affect household electricity demand.

### 2.2. Heterogeneous Effects

Residential electricity consumption may have different patterns among households which have different incomes or live in urban or rural areas. We examine these two heterogeneous effects by estimating (2) and (3) as follows:

(2) ln⁡Eit=θ0+θ1·ln⁡(PE_resp)×Richit+θ2·ln⁡(PE_resp)+θ3·Richit+θ4·ln⁡(PE_ngp)+θ5·Zit+φt+εit,

where Rich_*it*_ is a dummy variable which equals one if the income of household *i* in year *t* is greater than the sample median. The interaction term between ln(PE_res_*p*_) and Richit captures the difference in price elasticity of the rich compared with the poor. Taking the poor household as the benchmark, the price elasticities of the poor household and the rich household are estimated by the coefficients *θ*_2_ and (*θ*_1_ + *θ*_2_), respectively. Consider the following:

(3) ln⁡Eit=γ0+γ1·ln⁡ PE_resp×Urbanit+γ2·ln⁡Incomeit×Urbanit+γ3·ln⁡PE_resp+γ4·ln⁡Incomeit+γ5·Urbanit+γ6·ln⁡PE_ngp+γ7·Zit+φt+εit,

where Urban_*it*_ is the dummy variable for urban households. Similarly, the interaction terms between the price/income variables and the urban dummy variable capture the differences in price and income elasticities of urban households compared with rural households. 

### 2.3. Estimation Issues

Econometrically estimating the electricity demand function presents several challenges. First, simultaneity problems exist between marginal price and consumption if aggregated data are used or consumers face a nonlinear price scheme because there is reverse causality between demand and price. Fortunately, the simultaneity problem is avoided in our study since we use the household level data in which the household is clearly the price taker. In addition, electricity prices have been highly regulated in China and local sales prices were set by provincial governments based on the costs of power generation, transmission, and distribution. The increasing block tariff did not start nationwide until July, 2012. During our study period (2008-2009), consumers faced the single price scheme, which enabled us to avoid the endogeneity problem caused by the nonlinear pricing scheme. Second, energy demand is influenced by long-term household decisions over appliance purchases and dwelling characteristics. Some studies have used a system of equations where the household makes two-stage optimization decisions. In the first stage, the short-run consumption of electricity depends, among other variables, on electricity price and income. In the second stage, the purchasing decision for durable goods, such as electronic appliances, is modeled [11, 12]. Since the system of equations approach has a very high data requirement (information on holdings of household-specific appliances and residence features), single equation specifications for household energy demand are most often used in linear or logarithmic form [13–17].

There are several options for estimating (1)–(3): pooled OLS, which assumes away significant individual or temporal effects among the panel; the fixed effects or random effects models, which assume there are unobserved specific individual and temporal effects. As introduced above, electricity price has been highly regulated in China. During our study period, the prices were quite uniform within a province and remained stable over years. A lack of variation in the price variable forced us to adopt the pooled OLS technique. 

## 3. Data and Estimation Results

### 3.1. Data

The household level data used in this study is provided by the China Family Panel Studies (CFPS) project, conducted annually by Peking University of China since 2008. In 2008 and 2009, the survey was carried out in Beijing, Shanghai, and Guangdong, which are located in eastern China. Stratified random sampling is applied, which ensures representativeness and randomness. A more detailed description of the CFPS project can be found in Hvistendahl [18]. The CFPS survey data contain information on various aspects of the household, such as socioeconomic information and demographic status, education information, and health information of the households. The CFPS survey data have been employed in a number of studies, such as Luo and Zhang [19] on health and labor market outcomes and Su and Heshmati [20] on gender wage difference.

A panel dataset is constructed using CFPS 2008 and 2009. In 2008, the survey covered 2,375 households, among which 1,940 households were followed up in 2009. Keeping the families that are observed in both years and with nonmissing electricity usage, the balanced panel covers 1,649 households.

Descriptive statistics are reported in Table 1. In our dataset, the average electricity consumption per capita is 37.78 kWh per month, which is 37% higher than the national average of 27.66 kWh per month [21]. (The average electricity usage for each household is 112.97 kWh per month in our dataset. On average there are 2.99 household members. So each person consumes 37.78 kWh per month.) This is because Beijing, Shanghai, and Guangdong, where the data were collected, are among the most developed provinces of China. As introduced above, a single price scheme for residential electricity was implemented in China during our investigating period. The nominal electricity prices in 2008 and 2009 for Beijing, Shanghai, and Guangdong remained unchanged at 0.47, 0.54, and 0.61 Yuan/KWH, respectively. The prices of electricity and natural gas are deflated using the provincial consumer price index (CPI).

### 3.2. Estimation Results

Columns (2)–(4) of Table 2 report the estimation coefficients and associated *t*-values for the three electricity demand models ((1)–(3)) based on the household data using the OLS technique. In model 1, the own price elasticity of electricity demand is estimated to be −2.477, which carries the expected sign and is statistically significant at the 1% level. This suggests that a 1% increase in residential electricity price would result in a more than 2% decline in household electricity demand (ceteris paribus). The result implies that households are very responsive to electricity price changes. Electricity consumption is responsive to the level of income with an income elasticity of 0.058. However, since elasticity is well below unity, income growth results in a much less than proportional increase in electricity consumption.

An examination of the coefficient on the price of natural gas that is included in the model provides the cross elasticity between electricity and natural gas. The estimated coefficient is −0.486 and marginally significant. The result implies that there is a weak complementary relationship between electricity and natural gas, which reflects that electricity and natural gas have largely independent uses and limited switching. A similar complementary relationship between electricity and natural gas has been found for California residents [22] and Indian residents [23].

As we expected, larger dwelling size and more family members increase the household consumption of electricity. A 1% increase in the number of square meters results in about a 0.162% increase in the household's demand for electricity, while increasing the family number by one results in a 6.8% increase in the household demand for electricity (ceteris paribus).

Finally, the demographic characteristics may also influence the electricity consumption of the household. A one-year increase in the period of education results in a 6.4% increase in electricity consumption (ceteris paribus).

In model 2, we add a dummy variable that represents relatively rich families and associated interaction terms to control for the effect of income difference on electricity demand and the estimation results are reported in column 3 of Table 2. As indicated above, the estimated coefficient of the interaction term ln(PE_res)*Rich can be interpreted as the difference in price responsiveness of electricity demand between the rich household and poor household. The estimated coefficient is small and statistically insignificant, which means that the rich family and the poor family respond similarly to electricity price changes. The estimated coefficients of other variables in model 2 remain stable compared with model 1. 

In model 3, we add a dummy variable that represents whether the family lives in an urban area or a rural area and the associated interaction terms controlling for the effect of living location on electricity demand. The estimation results are reported in Column 4 of Table 2. The price elasticity for rural and urban households is −3.735 and −1.091, respectively, while the income elasticity is 0.063 and 0.023, respectively. These results indicate that urban residential electricity consumption is less elastic to both price and income. The estimates provide supportive evidence that urban household electricity consumption is less sensitive to price and income changes. Raising electricity price will be more detrimental to rural families. The income increase of the rural households will lead to more residential electricity demand. However, the overall effect is mild.

## 4. Conclusions

 The paper provides the results of the econometric estimation of the electricity demand model using a dataset consisting of information at the individual household level in China. The basic model is used to determine the responsiveness of electricity consumption to its own price, income, price of alternative fuel, and variables relating to the social-economic characteristics of the household. The estimated models demonstrate the importance of including the household level information, which is impossible using aggregate level data. Demand elasticities for the heterogeneous household level are also examined. 

We found that residential demand for electricity responds rather sensitively to its own price in China. There might be two reasons to explain the seemingly high estimate of our study. First, the estimates of electricity price elasticity depend on the model specification and data. Taylor [24] reviewed many studies and found electricity to be much more price elastic in the long run. Our estimated results, using a pooled two-year dataset and OLS technique, are consistent with his conclusion. Second, China's economy has undergone rapid development and modernization during the past 30 years. Most Chinese residents have experienced the dramatic change from lacking the basic living necessities before the 1980s to, today, being able to afford the costly electrical appliances (such as air conditioners, shower heaters, and washing machines) which represent the modern life style. However, for the Chinese, the virtue of thrift and the preference to save have hardly changed. It could be expected that an increase in the price of electricity might result in many households cutting back the use of electric appliances, for instance, they may choose to wash their clothes by hand, instead of using a washing machine, or to turn on the air-conditioner for only a few hours on a hot day instead of running it all day. Supportive evidence is that high estimates of the price elasticity of residential electricity demand were found in studies in the US before the 1970s [25, 26] and in some developing countries or regions, such as Taiwan [27] and Honduras [28]. 

From the policy point of view, the high price elasticity of residential electricity demand implies that there is the potential to use the price instrument to conserve electricity consumption. Electricity pricing schemes, such as the increasing block tariff, seasonal pricing, and/or residential time-of-use pricing, will increase the overall electricity price level and thus can effectively curb the demand of the electricity.

Since July 1st, 2012, China has begun to implement the increasing block tariff nationwide. How to design an effective tariff structure to ensure that the new pricing scheme can improve efficiency and equity is an essential question. The keys to the increasing block tariff design include the number of blocks and the volume and price in different blocks. The examination of the electricity demand elasticity of different groups shows that the high income group is more price elastic than the low income group, while rural families demonstrate more price elasticity than urban families. These results have three implications for designing the increasing block tariff. First, to ensure equity and satisfy the basic needs of consumers, the electricity volume of the first block should be set large enough to cover the usage of most residents. Second, the price of the last block, which targets the high income households, should be set high to ensure an effective cut in consumption. Third, since our results also show that raising electricity prices will be more detrimental to rural families, it is necessary to separate the rural and urban households and design different volumes and prices in each block for each group. 

Finally, as would be expected, the estimates for income elasticities show that electricity is a necessity. The relatively low value of this elasticity means that residential electricity consumption would grow moderately with further growth in the Chinese residents' incomes.

## Figures and Tables

**Table 1:** Descriptive statistics of the household survey data.

Variable	Description	Obs.	Mean	Std. dev.	Min.	Max.
E	Electricity usages (KWh/family/month)	3,298	112.97	88.65	0	550
Income^a^	Household income (Yuan)	3,298	37,239.13	43,856.17	0	33,8000
HSIZE^a^	Dwelling area (m^2^/family)	3,298	92.20	70.45	0	800
FSIZE	Family size (person/family)	3,298	2.99	1.33	1	12
PE_res	Residential electricity price (Yuan/KWh)	3,298	0.56	0.06	0.49	0.62
PE_ind	Industrial electricity price (Yuan/KWh)	3,298	0.82	0.04	0.76	0.89
PE_ng	Natural gas price (Yuan/m^3^)	3,298	6.12	0.62	5.50	7.29
Age	Age of household head	2,072	59.04	13.19	17	95
Edu	Years of education of household head	2,704	8.21	4.36	1	19

Note: ^a^For income, HSIZE, age, and edu, we replace missing values with 0 and create missing indicators for missing observations. Missing indicators are controlled for in the following regressions.

**Table 2:** Estimation results using OLS technique.

	Model 1: basic	Model 2: income effect	Model 3: urban versus rural
Dependent variable: ln(residential electricity consumption)			
ln(PE_res)	−2.477***	−2.638***	−3.735***
	(0.374)	(0.493)	(0.492)
ln(Income)	0.058***	0.016**	0.063***
	(0.005)	(0.007)	(0.009)
ln(PE_ng)	−0.486**	(0.252)	0.304
	(0.247)	(0.258)	(0.246)
ln(HSIZE)	0.162***	0.103***	0.159***
	(0.021)	(0.030)	(0.029)
FSIZE	0.068***	0.069***	0.099***
	(0.013)	(0.013)	(0.012)
Age	0.002	0.000	−0.005***
	(0.001)	(0.001)	(0.001)
Edu	0.064***	0.052***	0.030***
	(0.004)	(0.005)	(0.003)
ln(PE_res)*Rich		0.482	
		(0.661)	
Rich		0.201	
		(0.291)	
ln(PE_res)*Urban			2.644***
			(0.652)
ln(Income)*Urban			−0.040***
			(0.011)
Urban			(0.240)
			(0.307)
Year FE	Y	Y	Y
	OLS	OLS	OLS
Observations	3298.000	3298.000	3298.000
R-squared	0.160	0.186	0.239

Note: Robust standard errors in parentheses; ***P < 0.01, **P < 0.05, *P < 0.1.
